# Association of Urinary Phthalate and Phthalate Replacement Metabolite Concentrations with Serum Lipid Biomarker Levels among Pregnant Women Attending a Fertility Center

**DOI:** 10.3390/toxics10060292

**Published:** 2022-05-28

**Authors:** Lidia Mínguez-Alarcón, Paige L. Williams, Tamarra James-Todd, Irene Souter, Jennifer B. Ford, Kathryn M. Rexrode, Antonia M. Calafat, Russ Hauser, Jorge E. Chavarro

**Affiliations:** 1 Channing Division of Network Medicine, Harvard Medical School & Brigham and Women’s Hospital, Boston, MA 02115, USA; jchavarr@hsph.harvard.edu; 2 Department of Environmental Health, Harvard T.H. Chan School of Public Health, Boston, MA 02115, USA; tjtodd@hsph.harvard.edu (T.J.-T.); jford@hsph.harvard.edu (J.B.F.); rhauser@hsph.harvard.edu (R.H.); 3 Department of Biostatistics, Harvard T.H. Chan School of Public Health, Boston, MA 02115, USA; paige@sdac.harvard.edu; 4 Department of Epidemiology, Harvard T.H. Chan School of Public Health, Boston, MA 02115, USA; 5 Massachusetts General Hospital Fertility Center and Harvard Medical School, Boston, MA 02114, USA; isouter@mgh.harvard.edu; 6 Division of Women’s Health, Department of Medicine, Brigham and Women’s Hospital, Harvard Medical School, Boston, MA 02115, USA; krexrode@bwh.harvard.edu; 7 National Center for Environmental Health, Centers for Disease Control and Prevention, Atlanta, GA 30329, USA; aic7@cdc.gov; 8 Department of Obstetrics, Gynecology and Reproductive Biology, Harvard Medical School, Boston, MA 02115, USA; 9 Departments of Nutrition and Epidemiology, Harvard T.H. Chan School of Public Health, Boston, MA 02115, USA

**Keywords:** phthalates, lipids, pregnancy

## Abstract

We examined whether urinary concentrations of phthalate and phthalate replacement metabolites were associated with lipid biomarker levels among pregnant women. This cross-sectional study included 175 women who enrolled in the Environment and Reproductive Health (EARTH) Study (2005–2017). We used linear regression models to assess the relationship between urinary phthalates and lipid biomarkers [triglycerides, total cholesterol, high density lipoprotein (HDL), non-HDL, and low-density lipoprotein (LDL) cholesterol] levels while adjusting for confounders. Pregnant women in the highest quartile of urinary mono(2-ethyl-5-carboxypentyl) phthalate (MECPP) had, overall, 14% [31 (95% CI = 6.56) mg/dL], 21% [33 (95% CI = 9.57) mg/dL] and 25% [30 (95% CI = 8.53) mg/dL] higher serum total, non-HDL and LDL cholesterol, respectively, compared to women in the lowest quartile of MECPP. Similar positive associations were found for urinary concentrations of other metabolites of di(2-ethylhexyl) phthalate, mono(2-ethylhexyl) phthalate, and mono(2-ethyl-5-oxohexyl) phthalate. Pregnant women with urinary mono-n-butyl phthalate (MBP) in the highest quartile had higher triglycerides and non-HDL cholesterol compared to women with MBP in the lowest quartile. Women with detectable concentrations of two phthalate replacement metabolites had lower HDL cholesterol compared to women with non-detectable concentrations. Gestational urinary concentrations of certain phthalate and phthalate replacement metabolites were associated with lipid levels among these women.

## 1. Introduction

Women with impaired fertility are at higher risk of cardiovascular disease (CVD) [1,2,3,4,5,6,7,8,9,10,11,12]. CVD is the leading cause of mortality in women as well as the leading cause of pregnancy-related mortality in the United States (US) [13]. Around 16% of the pregnancy-related deaths in the US between 2014 and 2017 were attributable to cardiovascular conditions. Dyslipidemia, characterized by high circulating levels of triglycerides, total cholesterol and low-density lipoprotein (LDL), in addition to lower circulating levels of high-density lipoprotein (HDL) cholesterol, is a well-known CVD risk factor and reflects the global obesity epidemic [14]. Numerous factors have been related to the rising prevalence of these important conditions, including exposure to phthalates, which have been called “obesogens” and can alter the endocrine system [15]. The Obesogen Hypothesis proposes that obesogens may cause obesity by disrupting the adipocyte metabolism, which may lead to altered metabolic outcomes or by dysregulating appetite and satiety. Everyday consumer products including food packaging and diet confer daily exposure to endocrine-disrupting “obesogens” [15].

Phthalates, many of which are considered endocrine-disrupting chemicals, can be used in a multitude of consumer products, leading to widespread general population exposure [16,17,18,19,20]. Exposure to phthalates occurs through ingestion, inhalation, and dermal absorption [21,22,23,24,25,26,27,28]. After entering the body, phthalates are quickly hydrolyzed to their respective monoester metabolites that are biologically active. For some phthalate diesters, secondary oxidative metabolites are also formed and comprise the greater proportion of measured metabolites. Although phthalates do not persist in the body and have relatively short biological half-lives (<24 h), exposure to phthalates is repeated, episodic, and chronic [29,30,31]. Urine is the optimal matrix for quantifying phthalates as a result of phthalates’ short half-lives and non-persistent nature, as well as for being a non-invasive and convenient medium for biological monitoring [32].

Di(2-ethylhexyl) phthalate (DEHP) is primarily added to plastics to make them flexible, and can be found in consumer products, flooring and wall coverings, food contact applications, and medical devices. Metabolites of DEHP include mono(2-ethylhexyl) phthalate (MEHP), mono(2-ethyl-5-hydroxyhexyl phthalate (MEHHP), mono(2-ethyl-5-oxohexyl) phthalate (MEOHP), and mono(2-ethyl-5-carboxypentyl) phthalate (MECPP). Parent compounds of other phthalate metabolites, such as mono-n-butyl phthalate (MnBP), mono-isobutyl phthalate (MiBP), and mono-benzyl phthalate (MBzP) can be found in personal care products and other consumer products [18,33]. Di(isononyl)cyclohexane-1,2-dicarboxylate (DINCH), a non-phthalate plasticizer, was introduced commercially in 2002 as a safer alternative to ortho-phthalate esters (e.g., DEHP) because of its more favorable toxicological profile [34]. The use of DINCH was approved by the European Food Safety Authority in 2006. DINCH, a replacement of DEHP in vinyl plastics, is used in many U.S. polyvinyl chloride (PVC) products, particularly in food packaging and in the manufacturing of toys, building materials and medical devices.

Among reproductive-aged women participating in the 2001–2010 NHANES, urinary concentrations of the DEHP metabolites were strongly associated with an increased risk in metabolic syndrome, including higher triglycerides and lower HDL [35]. Pregnant women represent a vulnerable study population to endocrine-disrupting chemical exposure, including phthalates, with potential consequences not only for maternal health but also to offspring health [36]. Yet, to the best of our knowledge, only one study has examined the association between urinary concentrations of phthalate metabolites and circulating lipid biomarker levels in pregnant women from the general population [37]. This study showed that concentrations of some phthalate metabolites were positively associated with circulating tryglicerides and total cholesterol during pregnancy. Due to the scarcity of available literature on the topic and the importance of studying cardiovascular risk factors among subfertile women, we investigated whether urinary concentrations of phthalates and phthalate replacements are associated with serum lipids measured in serum among pregnant women attending a fertility center.

## 2. Materials and Methods

### 2.1. Study Population

We evaluated women enrolled in the Environment and Reproductive Health (EARTH) Study, a prospective cohort established to assess environmental and dietary determinants of fertility at the Massachusetts General Hospital (MGH) Fertility Center [38]. Women between 18 and 45 years old, using their own gametes for fertility treatment at the MGH Fertility Center, were eligible to participate; approximately 60% of those contacted by the research staff enrolled. For this specific analysis, we included 175 women participating in EARTH with data on both urinary metabolites of phthalates and phthalate replacements, in addition to serum lipid biomarker levels during pregnancy. Compared to excluded women also participating in the EARTH Study, included participants in this analysis were most likely to undergo IVF fertility treatments (compared to IUI and also non-medically pregnancies), and have a female factor infertility diagnosis (compared to male factor and unexplained fertility). Participants’ dates of birth were collected at entry, and weight and height were measured by trained study staff. Body mass index (BMI) was calculated as weight (in kilograms) divided by height (in meters) squared at enrollment. At this same time point, research staff administered sociodemographic, lifestyle and medical history questionnaires to participants, which self-reported important covariate information. Study participants also completed a comprehensive questionnaire on family, medical, reproductive, and occupational history; consumer products use, smoking history, and physical activity. Women were specifically asked about physical activity, frequency of tobacco, alcohol and illicit substance use, personal care and household product use, parental comorbidities, maternal smoking during pregnancy, and medical diagnoses of comorbidities, among others. Infertility diagnosis was assigned by physicians using the Society of Assisted Reproductive Technology definitions (SART). Pregnancy covariates were abstracted from electronic medical records. The study was approved by the Human Subject Committees of the Harvard T.H. Chan School of Public Health, MGH, and the Centers for Disease Control and Prevention (CDC). Participants each signed an informed consent document after the study procedures were explained by trained research study staff, and after all the participants’ questions were answered.

### 2.2. Exposure Assessment

During pregnancy, each woman collected up to three spot urine samples (one per trimester) in a sterile polypropylene specimen cup. For this analysis, we included one urine sample per woman—the one that was collected the same day as the blood sample, in order to measure lipid biomarkers in serum. Specific gravity (SG) was measured at room temperature using a handheld refractometer (National Instrument Company, Inc., Baltimore, MD, USA) calibrated with deionized water before each measurement. As a result of the potential for bias, rather than correcting by SG, we instead used the unadjusted urinary metabolite concentrations and adjusted for SG by including this as a covariate in the statistical models [39,40]. The urine was stored at −80 °C, and samples were shipped on dry ice overnight to the CDC for analysis. Following strict quality control/quality assurance practices, as previously described [41,42], we used online solid-phase extraction coupled with isotope dilution-high-performance liquid chromatography-tandem mass spectrometry in order to quantify the urinary concentrations of eight phthalate metabolites (monoethyl phthalate (MEP), MBP, MiBP, MBzP, MEHP, MEHHP, MEOHP, MECPP), and two metabolites of DINCH, a phthalate replacement [cyclohexane-1,2-dicarboxylic acid monohydroxy isononyl ester (MHiNCH) and cyclohexane-1,2-dicarboxylic acid monocarboxyisooctyl ester (MCOCH)]. Limits of detection (LOD) ranged from 0.1 to 1.2 µg/L.

### 2.3. Outcome Assessment

Women participating in the study provided up to three blood samples (5 mL) during pregnancy. Serum samples were aliquoted, frozen, and stored at −80 °C until transfer to the Clinical and Epidemiologic (CER) Laboratory at the Boston Children’s Hospital (Boston, MA, USA). In one serum sample per female participant randomly selected, we simultaneously analyzed three different lipid biomarkers, including triglycerides [43], total cholesterol [44], and HDL cholesterol levels (mg/dL), with the Roche Cobas 6000 system, using reagents and calibrators from Roche Diagnostics (Indianapolis, IN, USA). These assays are approved by the Food and Drug Administration for clinical use. The CERLab is certified by the Centers for Disease Control and Prevention/National Heart, Lung, and Blood Institute Lipid Standardization Program. We calculated non-HDL as the difference between total cholesterol and HDL cholesterol. We calculated LDL cholesterol following the Friedewald formula: LDL cholesterol = (total cholesterol) − (HDL cholesterol) − (triglycerides/5) [45]. Triglycerides are determined with day-to-day reproducibilities of 1.8% and 1.7%. The coefficients of variation (CVs) for total cholesterol concentrations are 1.7% and 1.6%. HDL-Cs are determined with day-to-day reproducibilities of 3.3% and 1.7%.

### 2.4. Statistical Analysis

We presented demographic and reproductive characteristics as well as serum lipid biomarker levels using median ± interquartile ranges (IQRs) or percentages. All phthalate and DINCH metabolite concentrations below the LOD were assigned a value equal to the LOD divided by the square root of 2. We calculated the molar sum of DEHP metabolites (∑DEHP) by dividing each DEHP metabolite concentration by its molecular weight (g/mol), and then summing ([MEHP × (1/278.34)] + [MEHHP × (1/294.34)] + [MEOHP × (1/292.33)] + [MECPP × (1/308.33)]). We also reported distribution of urinary concentrations of phthalate and phthalate replacement metabolites using percentiles and means ± standard deviations (SDs). We divided the urinary phthalate metabolite concentrations in quartiles, and we used them as categorical exposure variables, with the lowest group considered as the reference group. Urinary MHiNCH and MCOCH were divided into two groups (detectable vs. non-detectable), since these metabolites have lower detection rates and smaller sample sizes than the other phthalate metabolites examined. Of note, for MEHP, we included all the values <LOD in the lowest category (31%), and we divided the remaining urinary concentrations into three groups based on their distribution. Kolmogorov–Smirnov test results documented that the serum lipid biomarker levels did not deviate substantially from a normal distribution. We used Spearman correlation coefficients (except for DINCH metabolites because of their relatively low detection rate) to assess correlations between the measured urinary metabolite concentrations as continuous variables. We used linear regression models to estimate the association between urinary phthalate and phthalate replacement metabolites, as categorical variables, and circulating lipid biomarker levels. In order to allow for better interpretation of the results, we presented population marginal means [46], adjusting for all the covariates in the model (at the mean level for continuous variables and for categorical variables at a value weighted according to their frequencies), and also translated each to a percentage increase or decrease for ease of interpretation.

Confounding was assessed using both prior knowledge regarding biological relevance, and descriptive statistics from our study population using directed acyclic graphs (DAGs). The variables considered as potential confounders included factors previously demonstrated to be related to phthalates or serum lipids, and factors associated with both urinary metabolites and serum lipid levels in this study [47,48]. We adjusted models for age at pregnancy (years), pre-pregnancy BMI (kg/m^2^), education (graduate degree vs. other), infertility diagnosis (female factor vs. other), cycle type (without medical treatment vs. other), number of babies (singleton vs. twins/triplets), trimester (first vs. second vs. third), and specific gravity. In order to evaluate the robustness of the findings, we conducted a sensitivity analysis that excluded pre-pregnancy BMI, as it could be a mediator of the phthalates on lipid measures; moreover, we stratified the main associations by trimester since lipid levels change across trimester during pregnancy. Statistical analyses were performed with SAS (version 9.4; SAS Institute Inc., Cary, NC, USA).

## 3. Results

### 3.1. Study Population Characteristics

The 175 women included in this analysis had a median (IQR) BMI of 22.3 (21.3, 25.9) kg/m^2^ at enrollment and age of 35 (33, 39) years during pregnancy when they provided the urine and serum samples included in this study (Table 1).

Subjects were predominantly white (88%), highly educated (60% had a graduate degree), and only 29% had ever smoked. The majority of the women became pregnant using fertility treatments (83% IUI+IVF), and 82% had singleton pregnancies. Both urine and blood samples were collected during the first, second, and third trimesters for 61 (35%), 47 (27%), and 67 (38%) women, respectively. Median (IQR) gestational ages (all in weeks) for women with samples collected in the first, second, and third trimesters were 8 (7, 9), 23 (20, 26), and 34 (33, 36), respectively. Median (IQR) triglycerides, total, HDL, and LDL cholesterol (all in mg/dL) were 181 (111, 251), 229 (189, 280), 68.0 (58.0, 79.0), and 120 (92.0, 158), respectively (Table 1).

### 3.2. Urinary Phthalate and Phthalate Replacement Measures

Detection frequencies for the urinary phthalate and phthalate replacement metabolites among women in this study were similar to those reported in U.S. adult females from the general population, with urine samples collected in similar years [20] (Table 2).

Detection frequency for urinary MEHP was moderate (69%), and for MHiNCH and MCOCH was relatively low (23% and 13%, respectively), whereas high detection frequency (>90%) was observed for the other phthalate metabolites. Urinary concentrations of phthalate and phthalate replacement metabolites measured in pregnancy samples among these women were comparable to those found in preconception samples among women in the same cohort study [49]. As expected, urinary concentrations of the DEHP metabolites (MEHP, MEHHP, MEOHP and MECPP) were highly correlated with each other (Spearman r ≥ 0.92) (Table S1). Urinary concentrations of other phthalate metabolites were weakly correlated with each other.

### 3.3. Main Results

In adjusted models, we observed associations between some urinary phthalate metabolite concentrations and pregnancy serum lipid levels, in terms of differences between women with metabolite concentrations in the highest compared to the lowest quartile (Table 3). Specifically, pregnant women in the highest quartile of MECPP had, overall, 14% [31 (95% CI = 6.56) mg/dL], 21% [33 (95% CI = 9.57) mg/dL] and 25% [30 (95% CI = 8.53) mg/dL] higher serum total, non-HDL, and LDL cholesterol, respectively, compared to women with MECPP concentrations in the lowest quartile. Similar positive associations were found for urinary concentrations of other DEHP metabolites such as MEHP and MEOHP. Pregnant women with urinary MBP concentrations in the highest quartile had higher serum triglycerides and non-HDL cholesterol than women with urinary MBP concentrations in the lowest quartile (adjusted means for Q4 vs. Q1 = 212 vs. 181 and 183 vs. 162 mg/dL, respectively). Despite the relatively small sample size and the low detection frequency for the two examined urinary DINCH metabolites, we observed that women with detectable urinary concentrations of each of the phthalate replacement metabolites, MHiNCH and MCOCH, had lower HDL cholesterol compared to women with non-detectable urinary concentrations (adjusted means for detectable vs. non-detectable = 67.1 vs. 74.6, and 66.2 vs. 77.6 mg/dL, respectively). No other clear patterns were observed (Table 3). After excluding pre-pregnancy BMI as a covariate, the model results were almost identical (data not shown).

### 3.4. Trimester-Specific Associations

Since lipid metabolism changes across trimester during pregnancy, we then conducted the analyses stratified by trimester (Table 4). We found that the observed associations were primarily observed for samples collected during the second trimester (median = 23 weeks of gestation), except for the two DINCH metabolites where the associations were mainly observed for samples obtained in the third trimester (median = 34 weeks). Associations for urinary MBP and non-HDL cholesterol, as well as MEHP and LDL-cholesterol, were also observed in samples collected during the first trimester (median = 8 weeks of gestation).

## 4. Discussion

In this cross-sectional analysis among women attending a fertility center, we found that women with higher urinary concentrations of some DEHP metabolites had higher serum total, non-HDL, and LDL cholesterol levels measured during pregnancy. We also observed positive associations of urinary MBP with serum triglycerides and non-HDL cholesterol levels, as well as inverse associations between the two examined urinary DINCH metabolites and serum HDL cholesterol. Associations remained after excluding BMI as a covariate, and were overall found in samples collected during the second trimester. These results suggest that pregnancy exposure to certain phthalates and phthalate replacements may be associated with dyslipidemia (characterized by high circulating levels of triglycerides, total cholesterol, and LDL, in addition to lower circulating levels of HDL cholesterol) during pregnancy among women participating in this cohort study. Among pregnant women from the same study cohort, we previously reported that urinary concentrations of MEP and MiBP were associated with higher pregnancy glucose, suggesting that exposure to certain phthalates may be a potentially modifiable risk factor of glucose dysregulation [50].

To the best of our acknowledge, only one epidemiologic study has assessed the relationship between urinary phthalate metabolites and circulating lipid biomarker levels during pregnancy [37]. In a cross-sectional analysis in which both urine and blood samples were collected at approximately 16 weeks of gestation, Vuong et al. reported that urinary MBzP was negatively associated with total cholesterol levels, and it was also an important contributor in the mixture models examining circulating triglycerides among 388 pregnant women participating in the Health Outcomes and Measures of the Environment (HOME) Study [37]. In a different study on pregnancy exposures in relation to post-natal lipids, Wu et al. prospectively investigated pregnancy urinary phthalate metabolite concentrations in relation to 4–8 years post-delivery lipid biomarker levels in 618 Mexican women [51]. The authors reported that the phthalate mixture was associated with lower HDL cholesterol and higher triglyceride levels. They also observed that the phthalate mixture was not associated with total and LDL cholesterol, however, urinary concentrations of MEP and the sum of metabolites of dibutyl phthalate (∑DBP) were both associated with lower and higher total cholesterol, respectively, and mono(2-ethyl-5-carboxypentyl) terephthalate and ∑DBP were associated with lower LDL cholesterol. Among pregnant women in our study, however, we found that some urinary phthalates and phthalate replacement metabolites were associated with higher overall dyslipidemia as measured by higher triglycerides, total, and LDL cholesterol, in addition to lower HDL cholesterol. Discrepancies in results among Wu et al. and our study may be due to differences in study population, number of urine samples collected, urinary phthalate metabolite concentrations (being much lower in our study), and timing for lipid assessment. While the epidemiologic literature on pregnancy exposure to phthalates and lipid profiles is still scarce, there is growing evidence that exposure to certain phthalates may affect circulating levels of some lipid biomarkers in women. Clearly, further epidemiologic studies including other study populations are warranted in order to clarify the directions of these associations.

Some phthalates [52,53,54] are known agonists of peroxisome proliferator-activated receptors (PPARs)-alpha, which also are key regulators of lipid metabolism [55]. Specifically, PPARs are involved in interpretation of fatty acid signals derived from dietary lipids, pathogenic lipoproteins, or essential fatty acid metabolites. Animal models have shown that exposure to DEHP can alter lipid levels by regulating hepatic energy metabolism through signaling of PPARs-alpha [56,57,58]. PPAR-alpha expression has been shown to be required for the toxicity of DEHP that results in altered plasma triglyceride levels. Other phthalate replacement biomarkers, such as MHiNCH, one of the metabolites of DINCH, are also capable of binding to PPARs, resulting in endocrine disruption and adipocyte cellular differentiation [59]. Epidemiologic studies have also demonstrated that exposure to phthalates can affect human health, observed as diminished reproductive function and higher pregnancy complications, increased risk of respiratory diseases, allergies, cardiometabolic diseases, and neurobehavioral disorders [60,61]. Variability in an individual’s exposure to phthalates can result from changes in the use of personal care products, diet, or daily activities, and exposure may vary over time [18]. Moreover, exposure to ortho-phthalates has declined over time, partially because their use has been restricted in many countries. Emerging plasticizers are replacing legacy phthalates in consumer products, and exposure to these replacements is expected to increase. Thus, additional studies in both animals and humans that explore the potential effects of these phthalate replacements on circulating lipid levels are also needed.

Limitations of this study include limited generalizability of these results to pregnant women in the general population, since this study includes subfertile women attending a fertility center. However, this study population is of public health importance, as women with impaired fertility are at higher risk of cardiovascular disease than other women [1,2,3,4,5,6,7,8,9,10,11,12]. Also, we did not exclude women with obesity and pregnancy complications, which may influence lipid metabolism in pregnant women. Secondly, the cross-sectional nature of this analysis does not allow us to establish that exposures occurred before the outcomes. Reverse causation is also possible, given the lipophilic nature of some phthalates [62]. Thirdly, as in any observational study, residual confounding by other chemical exposures, lifestyle, and reproductive factors is still possible. Particular interest for future studies should consider unmeasured confounding from diet and food packaging on the DEHP/replacement phthalate metabolite associations [62]. Fourthly, misclassification of the exposure is possible, especially for high molecular weight phthalates, given their short half-lives [18] and episodic nature of exposures to them. Fifthly, not all the collected blood samples were fasting, and this may impact the observed results. Sixthly, the observed results for MHiNCH and MCOCH, the two DINCH metabolites, should be taken cautiously given the relatively low detection frequency of these biomarkers in urine samples from pregnant women in this study. Despite these limitations, there are several strengths. Firstly, we evaluated two phthalate replacement metabolites, in addition to several phthalate metabolites. Secondly, we evaluated this research question using a well-established cohort of subfertile women at high risk of cardiovascular disease [6]. Evaluating exposures to prevalent EDCs as they relate to lipid levels in this subfertile population may provide key information that could aid in formulating recommendations for improving cardiometabolic health in this high-risk group.

## 5. Conclusions

To conclude, we observed positive associations between some DEHP metabolites and MBP and serum total, non-HDL, and LDL cholesterol levels measured among pregnant women seeking fertility care. We also observed negative associations between two metabolites of DINCH, a phthalate replacement, and serum HDL cholesterol. These results suggest that exposure to certain phthalates and phthalate replacements may be associated with dyslipidemia, a leading cause of cardiovascular and metabolic disease. Considering the scarce literature on the topic, further epidemiologic studies, including larger sample sizes, are warranted in order to explore phthalate mixtures as well as trimester-specific windows of exposure.

## Figures and Tables

**Table 1:** Demographic and reproductive characteristics as well as serum lipid biomarker levels [median (IQR) or N (%)] among 175 pregnant women in the Environment and Reproductive Health (EARTH) Study.

**Enrollment**	
Age, years	35.0 (32.0, 37.0)
Race, N (%)	
White	154 (88)
Black	5 (2)
Asian	8 (5)
Other	8 (5)
Body Mass Index, kg/m^2^	22.3 (21.2, 25.7)
Ever smoked, N (%)	50 (29)
Graduate degree, N (%)	105 (60)
Primary Infertility diagnosis, N (%)	
Male factor	58 (33)
Female factor	59 (33)
Unexplained	58 (33)
**Pregnancy**	
Age, years	35.0 (32.0, 38.0)
Cycle type, N (%)	
Without medical treatment	29 (17)
IUI	45 (26)
IVF	101 (57)
Number of babies, N (%)	
Singleton (1)	144 (82)
Twins (2)	28 (16)
Triplet (3)	3 (2)
Trimester of sample collection, N (%)	
1st	61 (35)
2nd	47 (27)
3rd	67 (38)
Triglycerides, mg/dL	181 (111, 251)
Total cholesterol, mg/dL	229 (189, 280)
HDL cholesterol, mg/dL	68.0 (58.0, 79.0)
Non-HDL cholesterol, mg/dL	161 (120, 206)
LDL cholesterol, mg/dL	120 (92.0, 158)

**Table 2:** Distribution of urinary phthalate and phthalate replacement metabolite concentrations (μg/L) among pregnant women in the Environment and Reproductive Health (EARTH) Study.

	N	Detection Frequency %	Maximum LOD (μg/L)	Mean (SD)	10th	25th	50th	75th	95th
Mono-n-butyl phthalate (MBP)	175	97	0.6	17.8 (31.6)	1.70	3.80	9.20	19.0	46.7
Mono-isobutyl phthalate (MiBP)	175	98	0.8	9.85 (15.4)	1.20	2.40	6.01	12.3	31.7
Monoethyl phthalate (MEP)	175	100	1.2	200 (544)	5.54	11.6	31.3	109	1500
Monobenzyl phthalate (MBzP)	175	93	0.3	7.54 (76.7)	0.40	0.90	2.50	5.80	24.9
Mono(2-ethylhexyl) phthalate (MEHP)	175	69	1.2	10.5 (50.0)	<LOD	<LOD	1.80	4.80	27.4
Mono(2-ethyl-5-hydroxyhexyl) phthalate (MEHHP)	175	99	0.7	48.6 (239)	1.40	2.90	7.00	18.3	115
Mono(2-ethyl-5-oxohexyl) phthalate (MEOHP)	175	98	0.7	31.5 (145)	1.10	2.50	5.60	14.0	56.5
Mono(2-ethyl-5-carboxypentyl) phthalate (MECPP)	175	100	0.4	59.1 (254)	2.80	5.50	11.7	28.2	104
Cyclohexane-1,2-dicarboxylic acid monohydroxy isononyl ester (MHiNCH)	111	23	0.4	0.97 (4.57)	<LOD	<LOD	<LOD	<LOD	1.40
Cyclohexane-1,2-dicarboxylic acid monocarboxyisooctyl ester (MCOCH)	83	13	0.5	0.68 (1.92)	<LOD	<LOD	<LOD	<LOD	1.10

Limit of detection (LOD); Standard deviation (SD).

**Table 3:** Adjusted^a^ serum levels of lipid biomarkers by groups of urinary phthalate and phthalate replacement metabolite concentrations among 175 pregnant women in the Environment and Reproductive Health (EARTH) Study.

	Triglycerides, mg/dL	Total Cholesterol, mg/dL	HDL Cholesterol, mg/dL	Non-HDL Cholesterol, mg/dL	LDL Cholesterol, mg/dL
MBP					
Q1	181 (157, 206)	234 (218, 250)	71.6 (66.6, 76.7)	162 (146, 178)	126 (112, 141)
Q2	211 (191, 231)ǂ	236 (222, 250)	69.6 (65.3, 73.9)	166 (153, 180)	124 (112, 137)
Q3	178 (157, 199)	228 (214, 242)	71.0 (66.6, 75.5)	157 (143, 171)	122 (109, 134)
Q4	212 (190, 234)ǂ	250 (235, 264)	66.3 (61.7, 70.9)	183 (169, 198)ǂ	141 (128, 154)
MiBP					
Q1	198 (174, 223)	246 (230, 262)	73.1 (68.0, 78.1)	174 (158, 189)	134 (120, 149)
Q2	214 (193, 235)	238 (225, 252)	69.1 (64.8, 73.4)	170 (156, 183)	127 (114, 140)
Q3	186 (164, 207)	238 (224, 252)	67.7 (63.3, 72.1)	171 (157, 185)	134 (121, 147)
Q4	185 (162, 209)	223 (208, 239)ǂ	68.6 (63.8, 73.5)	155 (140, 170)	118 (104, 132)
MEP					
Q1	207 (185, 230)	235 (220, 250)	66.5 (61.9, 71.1)	169 (154, 183)	127 (114, 140)
Q2	206 (185, 227)	251 (238, 265)	69.9 (65.6, 74.2)	182 (168, 195)	140 (128, 153)
Q3	188 (167, 209)	232 (218, 245)	69.9 (65.6, 74.1)	162 (149, 175)	124 (112, 136)
Q4	183 (160, 205)	230 (216, 244)	72.2 (67.7, 76.7)	158 (144, 172)	121 (108, 134)
MBzP					
Q1	185 (161, 209)	236 (220, 252)	73.5 (68.4, 78.4)	162 (147, 178)	125 (111, 140)
Q2	217 (196, 237)	230 (216, 243)	65.0 (60.8, 69.1) *	165 (152, 178)	122 (109, 134)
Q3	193 (172, 215)	245 (231, 260)	71.0 (66.5, 75.3)	174 (160, 189)	136 (123, 149)
Q4	186 (165, 209)	237 (223, 252)	69.7 (65.2, 74.2)	168 (153, 182)	130 (117, 144)
MEHP					
G1	190 (170, 211)	227 (214, 240)	68.1 (63.9, 72.3)	159 (146, 171)	121 (109, 133)
G2	200 (176, 224)	232 (217, 246)	71.3 (66.4, 76.3)	160 (146, 176)	121 (107, 135)
G3	182 (159, 206)	230 (215, 246)	70.2 (65.4, 75.1)	160 (145, 175)	124 (110, 138)
G4	209 (187, 230)	256 (242, 270)*	69.7 (65.2, 74.2)	186 (172, 200) *	145 (132, 157) *
MEHHP					
Q1	196 (172, 220)	238 (222, 254)	70.9 (65.9, 75.9)	167 (152, 183)	128 (114, 142)
Q2	183 (163, 204)	228 (215, 241)	70.3 (66.1, 74.6)	158 (145, 171)	122 (109, 133)
Q3	202 (181, 224)	228 (215, 242)	68.7 (64.3, 73.1)	160 (146, 173)	119 (107, 132)
Q4	202 (179, 225)	253 (238, 268)	68.6 (63.9, 73.3)	185 (170, 199)	144 (131, 158)
MEOHP					
Q1	184 (160, 207)	232 (216, 247)	69.6 (64.7, 74.4)	162 (147, 177)	126 (112, 140)
Q2	189 (168, 210)	229 (216, 243)	72.8 (68.5, 77.2)	157 (143, 170)	119 (107, 131)
Q3	201 (180, 222)	230 (217, 244)	67.1 (62.8, 71.3)	163 (150, 176)	123 (111, 135)
Q4	210 (186, 233)	255 (240, 270)ǂ	69.2 (64.3, 74.0)	186 (171, 201) *	144 (130, 158) ǂ
MECPP					
Q1	191 (166, 215)	225 (210, 241)	69.4 (64.3, 74.5)	156 (141, 172)	118 (104, 132)
Q2	193 (171, 214)	239 (225, 252)	72.2 (67.8, 76.6)	167 (153, 180)	128 (116, 140)
Q3	196 (175, 217)	226 (213, 240)	69.7 (65.4, 74.0)	157 (144, 170)	118 (106, 130)
Q4	204 (180, 228)	256 (240, 271) *	67.2 (62.3, 72.1)	189 (174, 205) *	148 (134, 162) *
∑DEHP					
Q1	129 (112, 147)	233 (217, 249)	71.4 (66.5, 76.4)	161 (146, 177)	125 (110, 139)
Q2	125 (110, 140)	236 (221, 249)	71.3 (66.9, 75.6)	164 (151, 177)	124 (111, 137)
Q3	125 (110, 141)	229 (215, 243)	67.5 (63.2, 71.9)	161 (148, 175)	123 (110, 135)
Q4	133 (115, 150)	250 (235, 266)	68.3 (63.5, 73.2)	182 (167, 197)ǂ	141 (127, 155)
MHiNCH					
G1 non-detectable	171 (143, 199)	220 (204, 236)	74.6 (69.3, 80.0)	145 (129, 161)	111 (96.1, 125)
G2 detectable	187 (172, 202)	223 (214, 231)	67.1 (64.2, 70.0) *	156 (147, 164)	118 (111, 126)
MCOCH					
G1 non-detectable	144 (109, 179)	223 (203, 244)	77.6 (70.7, 84.4)	146 (126, 165)	117 (98.5, 135)
G2 detectable	174 (161, 188)	216 (208, 224)	66.2 (63.6, 68.9) *	150 (142, 157)	115 (108, 122)

^a^ Data are presented as predicted marginal means (95% CI) unless otherwise noted, adjusted for age at pregnancy, pre-pregnancy BMI, education, infertility diagnosis, cycle type, number of babies, trimester, and specific gravity. * *p*-value < 0.05 when comparing that group with the lowest group. ǂ *p*-value < 0.10 when comparing that group with the lowest group. Mono-n-butyl phthalate (MBP); mono-isobutyl phthalate (MiBP); monoethyl phthalate (MEP); monobenzyl phthalate (MBzP); mono(2-ethylhexyl) phthalate (MEHP); mono(2-ethyl-5-hydroxyhexyl) phthalate (MEHHP); mono(2-ethyl-5-oxohexyl) phthalate (MEOHP); mono(2-ethyl-5-carboxypentyl) phthalate (MECPP); cyclohexane-1,2-dicarboxylic acid monohydroxy isononyl ester (MHiNCH); cyclohexane-1,2-dicarboxylic acid monocarboxyisooctyl ester (MCOCH). ∑DEHP = MEHP, MEHHP, MEOHP and MECPP.

**Table 4:** Adjusted ^a^ trimester-specific associations of urinary phthalate and phthalate replacement metabolite concentrations with serum levels of lipid biomarkers among 175 pregnant women in the Environment and Reproductive Health (EARTH) Study.

	Trimester 1 Median 8 Weeks (N = 61)	Trimester 2 Median 23 Weeks (N = 47)	Trimester 3 Median 34 Weeks (N = 67)
MBP	Triglycerides, mg/dL		
Q1	88.7 (61.5, 116)	159 (121, 1970)	262 (221, 304)
Q2	120 (91.2, 148)	183 (158, 209)	323 (288, 358)
Q3	116 (93.6, 139)	182 (154, 209)	231 (185, 277)
Q4	116 (96.5, 136)	208 (182, 235) ‡	306 (250, 360)
	Non-HDL cholesterol, mg/dL		
Q1	101 (87.8, 114)	132 (104, 161)	216 (188, 244)
Q2	113 (98.8, 127)	171 (152, 190) *	211 (187, 235)
Q3	120 (108, 131) *	173 (152, 194) *	190 (159, 222)
Q4	117 (107, 127) ǂ	196 (176, 217) *	244 (207, 282)
MEHP	Total cholesterol, mg/dL		
G1	184 (170, 199)	221 (198, 244)	271 (249, 293)
G2	192 (173, 209)	228 (202, 255)	288 (261, 314)
G3	168 (152, 183)	246 (229, 264) *	271 (233, 311)
G4	173 (162, 184)	277 (259, 296) *	325 (286, 364)*
	Non-HDL cholesterol, mg/dL		
G1	121 (109, 134)	144 (120, 167)	201 (179, 223)
G2	118 (103, 134)	159 (132, 1860	214 (188, 240)
G3	103 (89.5, 116) ǂ	171 (153, 188) *	198 (160, 237)
G4	111 (102, 121)	197 (179, 217) *	262 (224, 300) *
	LDL cholesterol, mg/dL		
G1	103 (91.1, 114)	112 (92.2, 132)	145 (125, 166)
G2	100 (85.9, 114)	124 (101, 146)	157 (132, 181)
G3	78.5 (66.5, 90.5) *	131 (116, 146)	154 (118, 190)
G4	87.1 (78.3, 95.9) ǂ	160 (144, 176) *	196 (161, 233)
MEOHP	Total cholesterol, mg/dL		
Q1	181 (166, 197)	213 (189, 237)	286 (258, 316)
Q2	179 (165, 193)	244 (220, 268) ‡	270 (245, 2940
Q3	168 (153, 182)	242 (224, 261) ‡	276 (248, 303)
Q4	178 (166, 191)	287 (265, 309) *	320 (281, 359)
	Non-HDL cholesterol, mg/dL		
Q1	115 (102, 128)	139 (116, 163)	218 (189, 247)
Q2	109 (97.2, 121)	167 (143, 191) ‡	196 (172, 221)
Q3	108 (95.5, 119)	165 (147, 184) ‡	207 (180, 235)
Q4	118 (108, 129)	*210 (189, 231)* *	248 (209, 287)
	LDL cholesterol, mg/dL		
Q1	100 (87.9, 112)	104 (84.1, 124)	164 (138, 190)
Q2	91.6 (80.5, 103)	132 (112, 153) *	139 (116, 161)
Q3	80.8 (69.7, 91.9) *	129 (113, 145) ǂ	151 (126, 176)
Q4	91.7 (82.1, 101)	170 (151, 188) *	192 (157, 228)
MECPP	Total cholesterol, mg/dL		
Q1	177 (163, 191)	213 (186, 240)	272 (240, 305)
Q2	185 (171, 200)	237 (213, 261)	285 (261, 308)
Q3	174 (160, 188)	240 (220, 260)	272 (244, 299)
Q4	175 (162, 187)	286 (263, 308) *	323 (284, 363)
	Non-HDL cholesterol, mg/dL		
Q1	112 (99.5, 124)	139 (112, 165)	203 (170, 235)
Q2	116 (104, 128)	163 (140, 186)	211 (187, 235)
Q3	110 (97.5, 122)	158 (140, 177)	204 (176, 232)
Q4	116 (105, 127)	211 (190, 233) *	255 (216, 294) ǂ
	LDL cholesterol, mg/dL		
Q1	92.4 (81.0, 104)	106 (82.9, 128)	148 (118, 177)
Q2	94.1 (82.3, 106)	126 (106, 146)	157 (135, 179)
Q3	86.6 (75.2, 98.0)	124 (108, 141)	145 (120, 171)
Q4	91.5 (81.2, 102)	170 (151, 188) *	197 (161, 233) ǂ
	Non-HDL cholesterol, mg/dL		
MHiNCH			
G1 non-detectable	103 (92.4, 114)	140 (114, 165)	176 (117, 234)
G2 detectable	114 (106, 121)	160 (146, 174)	199 (183, 215)
	Non-HDL cholesterol, mg/dL		
MCOCH			
G1 non-detectable	109 (93.4, 124)	163 (152, 174)	149 (107, 189)
G2 detectable	110 (104, 116)	195 (134, 256)	196 (182, 211) *

^a^ Data are presented as predicted marginal means (95% CI) unless otherwise noted, adjusted for age at pregnancy, pre-pregnancy BMI, education, infertility diagnosis, cycle type, number of babies, and specific gravity. * *p*-value < 0.05 when comparing that group with the lowest group. ‡ *p*-value < 0.10 when comparing that group with the lowest group. Mono-n-butyl phthalate (MBP); mono-isobutyl phthalate (MiBP); monoethyl phthalate (MEP); monobenzyl phthalate (MBzP); mono(2-ethylhexyl) phthalate (MEHP); mono(2-ethyl-5-hydroxyhexyl) phthalate (MEHHP); mono(2-ethyl-5-oxohexyl) phthalate (MEOHP); mono(2-ethyl-5-carboxypentyl) phthalate (MECPP); cyclohexane-1,2-dicarboxylic acid monohydroxy isononyl ester (MHiNCH); cyclohexane-1,2-dicarboxylic acid monocarboxyisooctyl ester (MCOCH).

## Data Availability

The data are not publicly available due to privacy and confidentiality reasons.

## Supplementary Materials

The following supporting information can be downloaded at: https://www.mdpi.com/article/10.3390/toxics10060292/s1, Table S1: Spearman correlations of urinary concentrations of phthalate metabolites among pregnant women in the Environment and Reproductive Health (EARTH) Study.
