# Supplementary material for: Association of Urinary Phthalate and Phthalate Replacement Metabolite Concentrations with Serum Lipid Biomarker Levels among Pregnant Women Attending a Fertility Center
Source: Toxics. 2022 May 28;10(6):292. doi: 10.3390/toxics10060292 (PMC9227340; doi:10.3390/toxics10060292)
Supplement: Supplementary file 1 [file toxics-10-00292-s001.zip › toxics-1713717-supplementary.pdf]

toxics

Article

Association of Urinary Phthalate and Phthalate Replacement Metabolite Concentrations with Serum Lipid Biomarker Levels among Pregnant Women Attending a Fertility Center

Lidia Mínguez-Alarcón [1,2,*], Paige L. Williams [3,4], Tamarra James-Todd [2,4], Irene Souter [5], Jennifer B. Ford [2], Kathryn M. Rexrode [6], Antonia M. Calafat [7], Russ Hauser [2,4,8] and Jorge E. Chavarro [1,4,9]

Table S1. Spearman correlations of urinary concentrations of phthalate metabolites among pregnant women in the Environment and Reproductive Health (EARTH) Study.

	MBP	MiBP	MEP	MBzP	MEHP	MEHHP	MEOHP	MECPP
**MBP**								
**MiBP**	0.30							
**MEP**	0.06	0.03						
**MBzP**	0.16	0.15	0.13					
**MEHP**	0.09	0.07	0.03	0.09				
**MEHHP**	0.12	0.08	0.07	0.13	0.99			
**MEOHP**	0.13	0.10	0.10	0.16	0.95	0.98		
**MECPP**	0.15	0.12	0.15	0.20	0.92	0.96	0.99	
